# Impact of Quality of Life on Survival and Mortality of Physically Independent Older Adults: Findings From Seven Years of Data

**DOI:** 10.7759/cureus.81719

**Published:** 2025-04-04

**Authors:** Mario Molari, Regina Célia Poli-Frederico, Denilson Castro Teixeira, Yshoner Antonio Silva-Diaz, Walter Sepulveda Loyola

**Affiliations:** 1 Associate Postgraduate Program in Physical Education, State University of Londrina, Londrina, BRA; 2 Postgraduation Program in Rehabilitation Sciences UEL-UNOPAR, State University of Londrina, Londrina, BRA; 3 Institute of Comprehensive Intercultural Health, Facultad de Ciencias de la Salud (FACISA) Universidad Nacional Toribio Rodríguez de Mendoza (UNTRM), Chachapoyas, PER; 4 Postgraduation Program in Rehabilitation Sciences UEL-UNOPAR, Health Sciences Center, State University of Londrina, Londrina, BRA; 5 Faculty of Health and Social Sciences, Universidad de Las Americas, Santiago, CHL

**Keywords:** aged, aging, mortality, physical function, quality of life

## Abstract

Introduction: Healthy aging emphasizes not only the extension of life expectancy but also the preservation of physical, mental, and social well-being, along with quality of life. However, several factors, such as the prevalence of chronic diseases and low educational and economic levels, may negatively affect the quality of life among older adults. This study aimed to identify the factors associated with a negative perception of quality of life and to evaluate the impact of negative quality of life on mortality over a seven-year follow-up period.

Methods: The research involved 419 older adults, 60 years or older, physically independent (levels 3 or 4 on the Spirduso Functional Status) and with mental status >17 points according to the Mini-Mental State Examination questionnaire. Variables assessed included sex, age group, education, economic class, quality of life index (QLI), race, marital status, falls in the last 12 months, body mass index (BMI), regular physical activity, and presence of comorbidities. Data on quality of life were collected using the Short Form Health Survey (SF-36), classifying in absolute binary threshold (positive/negative) into three variables: Health-Related Quality of Life (HRQoL), Quality of Life - Components of Physical Health (QL-CPH), and Quality of Life - Components of Mental Health (QL-CMH). Mortality information and causes of death from 2009 to 2016 were obtained from the Municipal Health Department. The association between negative perception of HRQoL with sociodemographic aspects, BMI, and comorbidities was analyzed using multivariate logistic regression analysis. To assess the association between quality of life variable and all-cause mortality, the Cox regression model was used, including crude and adjusted models. Kaplan-Meier curves and Mantel-Cox analysis were performed to compare survival curves between those subjects with positive and negative perceptions of quality of life.

Results: Older adults with negative HRQoL were characterized by lower educational levels, economic status, QLI scores, overweight status, and the presence of more than three comorbidities (p<0.01 for all). Negative HRQoL was associated with gender (male), low QLI, the presence of more than three comorbidities, and specific conditions, including hypertension, diabetes, neurological disorders, and rheumatic diseases (odds ratio (OR) ranging from 1.81 to 9.05). The adjusted Cox regression analysis showed that negative HRQoL (hazard ratio (HR) = 1.852; 95% confidence interval (CI): 1.05 to 3.27; p=0.034) and negative QL-CPH (HR = 1.83; 95% CI: 1.06 to 3.15; p=0.031) increased the risk of mortality in older adults. Kaplan-Meier survival curves indicated that older adults with a negative HRQoL index had a 21.3% probability of death over a seven-year period (p<0.01).

Conclusion: Sociodemographic factors, as well as the number and type of comorbidities, are associated with negative HRQoL in community-dwelling older adults. Furthermore, individuals with a negative perception of their quality of life face an increased risk of mortality.

## Introduction

Healthy aging has emerged as one of the primary goals of various international organizations, and it is the focus of the World Health Organization’s work on aging between 2015 and 2030 [1,2]. WHO defines healthy aging as “the process of developing and maintaining the functional ability that enables well-being in older age” [3]. The concept of healthy aging emphasizes not only the extension of life expectancy but also the preservation of physical, mental, and social well-being, enabling individuals to maintain their independence and social participation in society and impact the quality of life [3].

Achieving successful healthy aging entails maintaining optimal quality of life until the end of life. The concept of quality of life is considered multifactorial due to its subjective approach, in which people have different perceptions and aspirations regarding their living conditions [1,3]. Furthermore, the quality with which one wishes to live does not solely depend on individual actions [4]; a significant portion also stems from public policies [2], planned by governmental organizations. These actions result in basic survival needs such as housing, food, education, health, and work [5]. Therefore, living with quality of life is a challenge faced by developing countries, where social and economic barriers are significant and health strategies are insufficient [2].

In developing countries, older adults face different serious issues due to the high prevalence of chronic diseases [6], low educational attainment, and low per capita income, contributing to an increased vulnerability and potentially reduced quality of life [7-9]. Thus, aging with quality is a desire not always achieved by older adults and can impact the risk of different adverse events such as disability or mortality [10]. Moreover, perceptions of quality of life vary among older adults due to differences in health status, social support, and psychological well-being. [11]. However, most existing studies have been conducted in high-income countries, where socioeconomic conditions and healthcare access differ substantially. As a result, there is a critical gap in the literature regarding the specific determinants of quality of life and its long-term consequences in older adults from low- and middle-income settings. Addressing this gap, the present study aimed to examine the factors associated with a negative perception of quality of life and assess its impact on mortality over a seven-year follow-up period.

## Materials and methods

Study design

This is an observational, analytical, longitudinal epidemiological study, with baseline data collected from 2009 to 2010 and mortality measured over a seven-year period from 2009 to 2016. Both phases were approved by the Research Ethics Committee of Universidade Norte do Paraná under opinions PP/0070/09 and PP1168693. The study is part of the "Study on Longevity and Aging" (EELO) project, developed in the municipality of Londrina, Paraná, by Universidade Norte do Paraná (UNOPAR).

Population and sample

From a population of 43,610 older adults residing in the urban area of the municipality of Londrina (Brazil) in 2009, a calculated representative sample of 414 individuals was determined, considering a sampling error of 5%. Taking into account potential losses, an additional 20% was added, resulting in a total of 518 participants. The final sample consisted of 419 older adults, as 99 did not meet the inclusion criteria. Participants were recruited from the registries of all 38 Basic Health Units (UBSs) in the urban zone of the municipality between 2009 and 2010. Sampling was defined through stratified random sampling based on the number of older adults per region (15% central, 27% north, 23% south, 19% east, and 16% west).

The inclusion criteria were being 60 years or older; being physically independent (levels 3 or 4 on the Spirduso Functional Status Scale) [12]; have cognitive status without functional decline (>17 points according to the Mini-Mental State Examination questionnaire) [9]; and residing in the urban community of Londrina. Older adults with severe dysfunctions of the respiratory, neurological, and musculoskeletal systems (amputation or prosthesis) were excluded from the study.

Instruments

For data collection, the following instruments were used: interviews, questionnaires, and a survey of mortality rates during this period. Below are the details of these tools.

Interviews and Questionnaires

For data collection and analysis, certain evaluation criteria were established.

a) Socioeconomic data: Semi-structured interviews and anthropometric measurements were conducted to gather information on gender, age group, education, economic class, quality of life index (QLI), race, marital status, falls in the past 12 months, BMI, regular physical activity, and presence of comorbidities.

b) The QLI was established based on the variables of gender (female, male), age group (60-69, 70+ years), education (low, high), and economic class (low, high).

c) Quality of life: The Short Form Health Survey (SF-36) questionnaire was used, which is a multidimensional instrument composed of 36 items encompassing eight domains: functional capacity (10 items), physical aspects (4 items), pain (2 items), general health status (5 items), vitality (4 items), social aspects (2 items), emotional aspects (3 items), and mental health (5 items). It presents a score ranging from 0 to 100 points, where zero corresponds to the worst general health status and 100 to the best [13]. In this study, an absolute binary threshold (positive/negative) was established to stratify the quality of life results of the older adults into three variables: Health-Related Quality of Life (HRQoL), Quality of Life - Components of Physical Health (QL-CPH), and Quality of Life - Components of Mental Health (QL-CMH). Quality of life variables (HRQoL, QL-CPH, QV-CMH) were classified as "negative" when SF-36 scores were ≤50 points, whereas scores above 50 were considered "positive" [13]. The HRQoL variable included all eight domains; the QL-CPH variable was composed of four domains (functional capacity, limitations in physical aspects, pain, and general health status), while the QL-CMH variable encompassed four domains (vitality, social aspect, limitations in emotional aspects, and mental health).

The quality of life variables (HRQoL, QL-CPH, QV-CMH) were associated with the QLI to provide a real picture of the living conditions of the older adults, as the quality of life measure is subjective and varies from person to person. Additionally, it results from a set of actions reflecting education, socioeconomic status, and the role of the older adults in society [14].

Assessment of Mortality

The mortality data and causes of death for the period from 2009 to 2016 were obtained from the Municipal Health Department of Londrina, with information sourced from the national computerized system of the Information and Mortality Nucleus.

Data analysis

The results related to sociodemographic information and comorbidities were presented according to the frequency of responses, and their bivariate comparison with the quality of life variable was analyzed using the chi-square test. The association between negative perception of HRQoL with sociodemographic aspects, BMI, and comorbidities was analyzed using multivariate logistic regression analysis. To assess the association between quality of life variable and all-cause mortality in the study sample, the Cox regression model was used, including crude and adjusted models. The adjusted model included gender, age, and comorbidities. Hazard ratios (HRs) with their 95% confidence intervals (95% CI) were calculated. In addition, the Kaplan-Meier curve was constructed to compare the survival curves between those subjects with positive and negative quality of life using the log-rank test (Mantel-Cox test). Kaplan-Meier curve was performed for mortality and quality of life variables (HRQoL, QL-CPH, and QL-CMH) stratified by negative and positive scores. The analyses were conducted using the IBM SPSS Statistics for Windows, Version 25 (Released 2015; IBM Corp., Armonk, New York, United States), with a 95% CI and a significance level of 5% (p<0.05).

## Results

Sample profile

A total of 419 individuals were included in this study. Sociodemographic characteristics, fall incidents within the past 12 months, BMI, physical activity participation, comorbidities, and seven-year mortality rates are summarized in Table 1. The comparison between these variables in individuals with different classifications of health-related quality of life is also presented in this table. Older adults with a negative HRQoL were more likely to be women (p < 0.01), aged over 70 years (p = 0.07), overweight (p < 0.01), have lower education levels (p < 0.01), and belong to a lower socioeconomic status (p < 0.01). Additionally, this group showed significantly lower QoL index (p < 0.01), had more than three comorbidities (p < 0.01), and experienced higher mortality rates (p = 0.01).

**Table 1:** Sociodemographic characteristics, fall incidents, BMI, physical activity, comorbidities, and mortality among older adults based on perceptions of health-related quality of life *Statistical significance p < 0.05; HRQoL: Health-related quality of life; QLI – Quality of Life Index; BMI: Body Mass Index

Variables		General (n=419)	HRQoL		p
			Negative (n=277)	Positive (n=142)	
Sex	Women	282 (67.3%)	200 (72.2%)	82 (57.7%)	<0.01*
	Men	137 (32.7%)	77 (27.8%)	60 (42.3%)	
Age range	60-69 years old	222 (53%)	138 (49.8%)	84 (59.2%)	0.07
	70+ years	197 (47%)	139 (50.2%)	58 (40.8%)	
Education	Low	384 (91.6%)	266 (96%)	118 (83.1%)	0.01*
	Raised	35 (8.4%)	11 (4%)	24 (16.9%)	
Economy class	Low	341 (81.4%)	238 (85.9%)	103 (72.5%)	0.01*
	Raised	78 (18.6%)	39 (14.1%)	39 (27.5%)	
QLI	Low	315 (75.2%)	230 (83%)	85 (52.1%)	<0.01*
	Intermediate	96 (22.9%)	44 (15.9%)	52 (31.9%)	
	High	8 (1.9%)	3 (1.1%)	5 (3.1%)	
Race	Whites	259 (61.8%)	171 (61.7%)	88 (62%)	0.96
	Non-white	160 (38.2%)	106 (38.3%)	54 (38%)	
Marital status	Married	215 (51.3%)	134 (48.4%)	81 (57%)	0.38
	Separated	40 (9.5%)	29 (10.5%)	11 (7.7%)	
	Widower	142 (33.9%)	98 (35.4%)	44 (31%)	
	Single	22 (53%)	16 (5.8%)	6 (4.2%)	
Falls	No	255 (60.9%)	163 (58.8%)	92 (64.8%)	0.23
	Yes	164 (39.1%)	114 (41.2%)	50 (35.2%)	
BMI	Underweight	41 (9.8%)	28 (10.1%)	13 (9.2%)	<0.01*
	Normal weight	150 (35.8%)	82 (29.6%)	68 (47.9%)	
	Overweight	228 (54.4%)	167 (60.3%)	61 (43%)	
Physical activity Regular	No	221 (52.7%)	150 (54.2%)	71 (50%)	0.42
	Yes	198 (47.3%)	127 (45.8%)	71 (50%)	
Comorbidities	Up to 3	203 (48.4%)	114 (41.2%)	89 (62.7%)	<0.01*
	> 3	216 (51.6%)	163 (58.8%)	53 (37.3%)	
Mortality in 7 years	Living	344 (82.1%)	218 (78.7%)	126 (88.7%)	0.01*
	Dead	75 (17.9%)	59 (21.3%)	16 (11.3%)	

The prevalence of comorbidities is reported in Table 2. Individuals with negative HRQoL presented more prevalence of hypertension (p=0.04), diabetes (p=0.03), neurological disorders (p=0.01), and rheumatic diseases (p<0.01) compared to those with positive HRQoL.

**Table 2:** Comparison between variables comorbidities in older adults with different health-related quality of life *Statistical significance p < 0.05; HRQOL – Health-related quality of life

Variables		General (n=419)	HRQOL		P
			Negative (n=277)	Positive (n=142)	
Hypertension	No	147 (35.1%)	87 (31.4%)	60 (42.3%)	0.04*
	Yes	272 (64.9%)	190 (68.6%)	82 (57.7%)	
Diabetes	No	306 (73%)	193 (69.7%)	113 (79.6%)	0.03*
	Yes	113 (27%)	84 (30.3%)	29 (20.4%)	
Dyslipidemia	No	142 (33.9%)	92 (33.2%)	50 (35.2%)	0.68
	Yes	277 (66.1%)	185 (66.8%)	92 (64.8%)	
Osteoporosis	No	58 (13.8%)	35 (12.6%)	23 (16.2%)	0.31
	Yes	361 (86.2%)	242 (87.4%)	119 (83.8%)	
Neurological disease	No	403 (96.2%)	262 (94.6%)	141 (99.3%)	0.01*
	Yes	16 (3.8%)	15 (5.4%)	1 (0.7%)	
Lung disease	No	356 (85%)	240 (86.6%)	116 (81.7%)	0.12
	Yes	63 (15%)	37 (13.4%)	26 (18.3%)	
Rheumatic disease	No	232 (55.4%)	165 (5.6%)	67 (47.2%)	<0.01*
	Yes	187 (44.6%)	112 (40.4%)	75 (52.8%)	
Vascular disease	No	235 (56.1%)	155 (56%)	80 (56.3%)	0.94
	Yes	184 (43.9%)	122 (44%)	62 (43.7%)	
Heart disease	No	319 (76.1%)	213 (76.9%)	106 (74.6%)	0.60
	Yes	100 (23.9%)	64 (23.1%)	36 (25.4%)	
Thyroid Problems	No	366 (87.4%)	244 (88.1%)	122 (85.9%)	0.52
	Yes	53 (12.6%)	33 (11.9%)	20 (14.1%)	

The association between sociodemographic variables, BMI, and comorbidities in the perception of quality of life among older adults is presented in Table 3. The univariate analysis showed that sex, the QLI, having more than three comorbidities, and the prevalence of some specific chronic diseases (hypertension, diabetes, neurological disorders, and rheumatic conditions) increased the risk of having the quality of life of the older adults. In the multivariate model, the variables associated with negative health-related quality of life were age, sex, QLI (the interaction between education level and socioeconomic class), BMI, and neurological diseases.

**Table 3:** Association between sociodemographic aspects, BMI, and comorbidities with Negative HRQoL in older adults *Statistical significance p < 0.05; HR – Hazard Ratio; CI – Confidence Interval; QLI – Quality of Life Index

Variables	Univariate Analysis			Multivariate Analysis		
	OR	CI (95%)	P	OR	IC (95%)	P
Age Group						
60-69 years old	1.0(ref.)	-	0.071	-	-	0.001
70 or more	1.45	0.96 - 2.19		1.88	1.18 – 2.98	
Gender						
Women	1.0(ref.)	-	0.003	-	-	0.019
Men	1.9	1.24 – 2.90		1.81	1.10 – 2.97	
QLI (Education + Economic Class)						
Low	1.0(ref.)					
Intermediate	4.51	1.05 – 19.28	0.042	4.97	1.05 – 23.38	0.042
Elevated	1.41	0.31 – 6.23	0.65	1.17	0.24 – 5.73	0.84
BMI						
Normal weight	1.0(ref.)	-	-	-	-	-
Underweight	0.56	0.26 – 1.16	0.12	0.44	0.19 – 1.00	0.052
Overweight	1.27	0.61 – 2.61	0.514	0.95	0.42 – 2.17	0.916
Comorbidities						
Up to 3	1.0(ref.)	-	0	-	-	0.269
> 3	2.4	1.58 – 3.63		1.39	0.77 – 2.53	
Prevalence of Hypertension						
No	1.0(ref.)	-	0.048	-	-	0.975
Yes	1.52	1.00 – 232		1	0.59 – 1.69	
Prevalence of Diabetes						
No	1.0(ref.)	-	0.032	-	-	0.209
Yes	1.69	1.04 – 2.74		1.46	0.80 – 2.64	
Prevalence of Neurological Diseases						
No	1.0(ref.)	-	0.044	-	-	0.041
Yes	8.07	1.05 – 61.74		9.05	1.09–74.82	
Prevalence of Lung Diseases						
No	1.0(ref.)	-	0.125	-	-	0.23
Yes	1.6	0.87 – 2.95		1.55	0.75– 3.19	
Prevalence of Rheumatic Diseases						
No	1.0(ref.)	-	0.001	-	-	0.097
Yes	2.06	1.35 – 3.14		1.54	0.92 – 2.59	
Serious Diseases of the Past						
No	1.0(ref.)	-	0.691	-	-	0.266
Yes	1.08	0.71 – 1.66		1.31	0.81 –2.11	

The distribution of deceased and surviving individuals among older adults with different perceptions of quality of life is presented in Table 4. Notably, the majority of those who died during the follow-up period were individuals with a negative quality of life perception, accounting for 78% of those with negative HRQoL and 74.7% of those with negative QL-CPH.

**Table 4:** Mortality after 7 years follow-up in individuals with different classification of quality of life *Statistical significance p < 0.05; HRQoL – Health-related quality of life; QL-CPH – Quality of Life – Components of Physical Health; QL-CMH – Quality of Life – Components of Mental Health

Variables		General (n=419)	Survivors (n= 344)	Dead (n=75)	p
HRQoL	Positive (>50)	142 (33.9%)	126 (36.6%)	16 (21.3%)	0.01*
	Negative (≤50)	277 (66.1%)	218 (63.4%)	59 (78.7%)	
QL-CPH	Positive (>50)	162 (38.7%)	201 (58.4%)	19 (25.3%)	<0.01*
	Negative (≤ 50)	257 (61.3%)	143 (41.6%)	56 (74.7%)	
QL-CMH	Positive (> 50)	191 (54.4%)	161 (46.8%)	30 (40%)	0.28
	Negative (≤ 50)	228 (45.6%)	183 (53.2%)	45 (60%)	

In Table 5, the evolution of the number of deaths and the risk and survival indices in each group of older adults is observed, classified into two response levels in the HRQoL variable (negative/positive) over the seven-year follow-up of the study. It is noted that the mortality risk for older adults whose perception of quality of life was "negative" increased considerably compared to those with a "positive" perception, rising from a 2% risk in the second year of follow-up to 21% in the seventh year. The group with a "positive" perception reached 11% in the seventh year of follow-up.

**Table 5:** Probability of death and survival of elderly participants in the study observed by the Kaplan-Meier method during the seven years of follow-up HRQoL: Health-Related Quality of Life

Follow-Up Days	HRQoL (Negative)			HRQoL (Positive)		
	Deaths (n)	Risk (%)	Survival (%)	Deaths (n)	Risk (%)	Survival (%)
1st year (365 days)	2	0.7	99.3	2	1.4	98.6
2nd year (730 days)	4	2.2	97.8	2	2.8	97.2
3rd year (1095 days)	9	5.4	94.6	1	3.5	96.5
4th year (1440 days)	9	8.7	91.3	1	4.2	95.8
5th year (1825 days)	10	12.3	87.7	4	7.0	93.0
6th year (2190 days)	10	15.9	84.1	3	9.8	90.8
7th year (2556 days)	15	21.3	78.7	3	11.3	88.7
Total	59			16		

**Figure 1:**
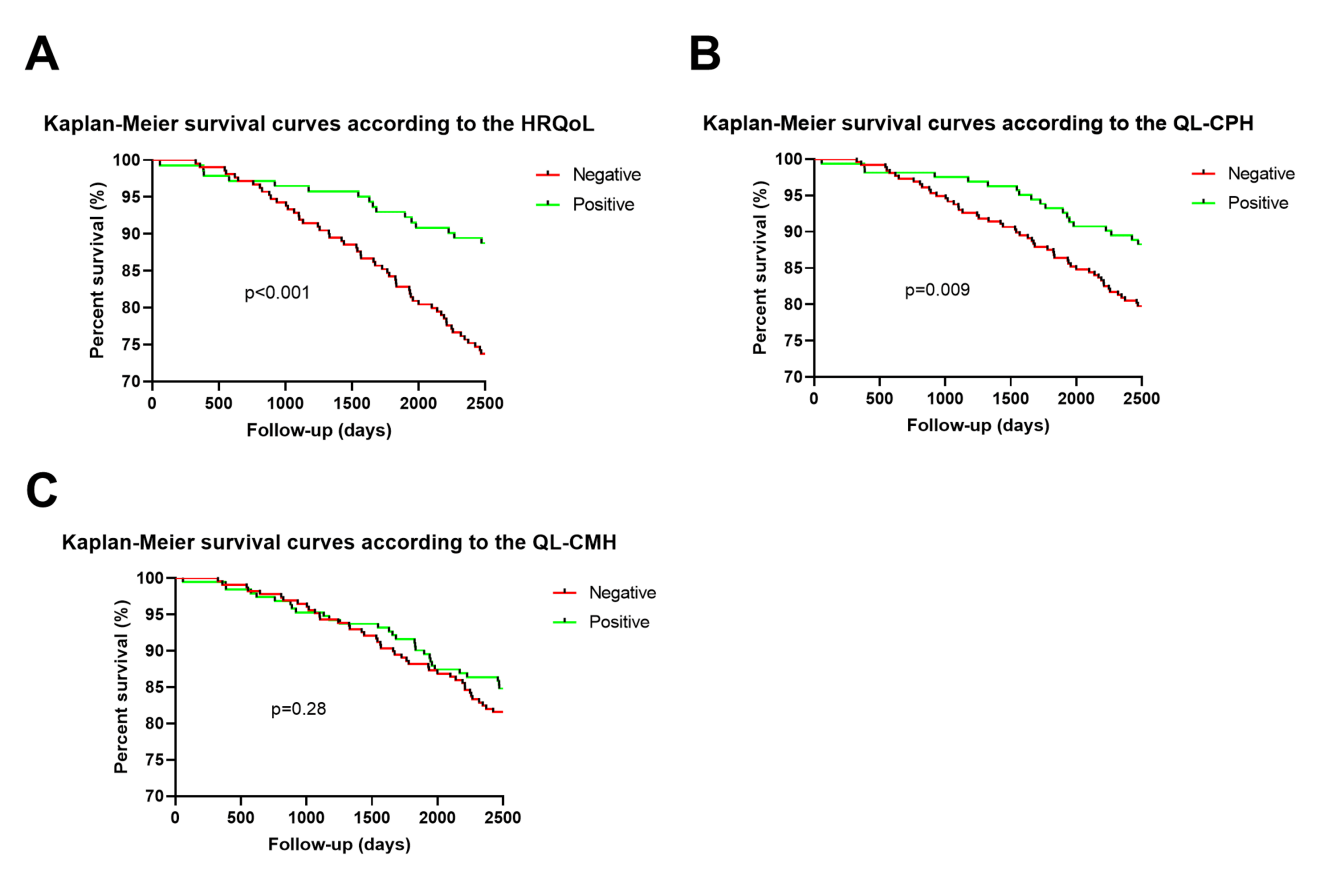
Kaplan-Meier survival curves for mortality and quality of life variables (HRQoL, QL-CPH, and QL-CMH) stratified by negative and positive scores HRQoL: Quality of Life Related to Health (composed of the eight domains of the SF36 - functional capacity, physical aspect limitation, pain, general health status, vitality, social aspects, emotional aspects limitation, and mental health). QL-CPH: Quality of Life - Components of Physical Health (composed of the four domains of the SF36 - functional capacity, physical aspect limitation, pain, general health status). QL-CMH: Quality of Life - Components of Mental Health (composed of the four domains of the SF36 - vitality, social aspects, emotional aspects limitation, and mental health)

The crude Cox regression analysis showed that negative HRQoL (HR=1.98; 95% CI: 1.14 to 2.24; p=0.015) and QL-CPH (HR=1.98; 95% CI: 1.17 to 3.31; p=0.011) increased the risk of mortality in older adults. Negative QL-CMH was not associated with risk of mortality (HR=1.28; 95% CI: 0.81 to 2.03; p=0.30). The adjusted Cox regression analysis showed that negative HRQoL (HR=1.852; 95% CI: 1.05 to 3.27; p=0.034) and QL-CPH (HR=1.83; 95% CI: 1.06 to 3.15; p=0.031) increased the risk of mortality in older adults. Negative QL-CMH was not associated with risk of mortality (HR=1.23; 95%CI: 0.77 to 1.98; p=0.39). The results of the Kaplan-Meier survival curves for mortality are presented according to the quality of life variables (HRQoL, QL-CPH, and QL-CMH) in Figures 1A-1C. The curves indicate that older adults with a "negative" score in the HRQoL variable have a 21.3% probability of death over a seven-year period (2556 days), while those with a "positive" score have an 11% probability (p<0.001). For QL-CPH, older adults with a "negative" score exhibit a 21% chance of dying, whereas those with a "positive" score have only 11% (p=0.009). In the case of the QL-CMH variable, the curves reveal that older adults with a "negative" score have a 19% probability of death, compared to 15% for those with a "positive" score, however, this was not statistically significant (p=0.28).

## Discussion

The aim of this study was to analyze the factors associated with a negative perception of quality of life and to evaluate the impact of poor quality of life on mortality among community-dwelling older adults over a seven-year follow-up period. The findings of this study underscore the critical relationship between the perception of quality of life and mortality among community-dwelling older adults over a seven-year follow-up period. Our analysis revealed that male gender, older age, lower income, lower educational levels, and a higher burden of chronic diseases were all significant factors associated with a negative perception of quality of life. Furthermore, a negative quality of life was strongly linked to an increased risk of mortality, reinforcing the importance of health perceptions as a predictor of long-term outcomes in older populations, particularly in developing countries such as Brazil, where regional disparities in social vulnerability persist [15,16].

The study observed that a negative perception of quality of life was significantly associated with mortality. This connection was particularly evident with components related to physical health (functional capacity, limitations in physical aspects, pain, and overall health status), which appeared to have a greater impact on negative perception and mortality. Previous studies support the notion that functional decline is a significant predictor of mortality among older adults [17,18]. Furthermore, physical health undergoes severe changes during the aging process, and when compounded by chronic diseases, these changes can further diminish their well-being [14]. Such deterioration leads to increased frailty and disability, placing older adults at greater risk of mortality [17,18].

Regarding the components of mental health (vitality, social aspect, limitations in emotional aspects, and mental well-being), there was no significant association with mortality. However, in this study, most older adults reported a negative perception in the domains of quality of life and mental health. This may be related to the losses faced in daily life, such as difficulties in performing daily activities, social distancing or problems in maintaining family ties and relationships, lack of opportunities to participate in community elder groups, limited financial independence, and feelings of not being respected by society, among other challenges that older adults encounter during the aging process [19].

In terms of comorbidities, hypertension, diabetes, neurological disorders, and rheumatic diseases, having more than three comorbidities was significantly associated with a negative quality of life. Chronic diseases are highly prevalent among older adults, particularly those over 70 years of age [10]. While many individuals adapt to their conditions by comparing their health to that of others in their social group, it is crucial to recognize that the aging process increases vulnerability to multiple health issues, which in turn affects overall quality of life [20]. This heightened vulnerability often leads to social isolation and a diminished capacity to engage in community life, both of which are detrimental to mental and physical health. Factors such as neglect, inadequate care, and a lack of ethical standards in elder care contribute to the social vulnerability of older adults [21]. Despite some older adults maintaining independence despite chronic conditions, prolonged exposure to adverse circumstances may eventually necessitate long-term care [22].

The persistence of chronic illness and the experience of its consequences lead older adults to perceive themselves through bodily changes, resulting in an assessment of their current health [23]. This influences whether they hold a positive or negative image of their quality of life. The internal subjective experience of the individual who is aware that personal well-being has been threatened and how the person responds to that experience [24]. This justifies the findings in our study, which indicate a higher number of older adults with a negative perception of their quality of life regarding the HRQoL, QL-CPH, and QL-CMH variables.

The current challenge for developed countries, which have a high older population, is to develop strategies to address health-related issues affecting this demographic [25]. While there is a significant number of older adults in both developed and developing countries, many developed nations have more advanced frameworks in place regarding research and government proposals. In other words, in developed countries, there is greater attention to the basic needs of older adults, which have emerged alongside the gradual increase in the population aged 60 and over [26]. Consequently, these countries can offer older adults better healthcare services and financial support for travel, enhancing their retirement experience and overall quality of life. European countries serve as examples of this, as they have been structuring themselves to meet this demand over the past 115 years - a timeframe necessary to ensure that older adults, despite making up a large portion of the population, have autonomy and a good quality of life [27].

Since older adults with a negative perception of quality of life demonstrated a higher risk of mortality and considering that some factors associated with negative quality of life - such as the presence of chronic diseases, socioeconomic status, and educational levels - are modifiable, this highlights the need for targeted interventions. These findings underscore the critical importance of focusing on these factors to inform and guide government policies aimed at improving the quality of life among older adults. In developing countries like Brazil, awareness of aging emerged nearly a century later, starting around the year 2000, when discussions arose regarding the situation of numerous Brazilians entering retirement [28]. However, the acceleration of longevity has been ongoing, and government proposals related to geriatrics and gerontology have not effectively promoted preparatory actions [29]. This includes the development of new programs for older adults, a more contemporary approach to retirement, and health initiatives that can enhance the quality of life for these individuals. Therefore, there is a need to adapt public policies to support various services for older adults and to devise strategies that generate a favorable economic balance. This will enable the establishment of new goals and action strategies that cater to this population, generating wealth to create a more conducive environment for older adults to age with better health and quality of life [30].

This study has several limitations that should be acknowledged. We did not collect data on medication use, healthcare access, psychosocial support, or causes of mortality. Additionally, quality of life was not assessed longitudinally. However, the study's strengths include a robust sample size, an extensive follow-up period, a comprehensive range of evaluated variables, and the use of the SF-36, a globally validated instrument. Despite assessing mortality over a seven-year period, not all variables were monitored longitudinally, which remains a key limitation. Future research should employ a longitudinal design incorporating a broader spectrum of clinical variables to deepen our understanding of the associations between quality of life, its temporal changes, and their impact on mortality and other adverse outcomes, such as hospitalization and disability. Moreover, exploring the biological mechanisms linking quality of life to mortality - particularly the differential impact of physical versus cognitive health - could provide valuable insights. Investigating the specific causes of mortality may also contribute to the development of more targeted interventions and public health strategies.

## Conclusions

We conclude that sociodemographic factors, as well as the number and type of comorbidities, are associated with a negative quality of life in community-dwelling older adults. In addition, a negative perception of their quality of life is associated with a high risk of mortality in this population. However, it is essential to understand that the approach to quality of life must be analyzed from a perspective that combines information related to education, income, and living conditions. This is because quality of life is multifactorial, varies from person to person, and alters an individual's perception of life based on the conflicts encountered in daily life.
